# Metabolomic profiling of finger millet: unlocking the secrets of a nutritious staple food

**DOI:** 10.3389/fpls.2025.1570787

**Published:** 2025-11-06

**Authors:** Vishal Gupta, Mahima Sharma, Sushil Kumar Gupta, Shaily Javeria, Zakir Amin, Suhail Ashraf, Javed Masood Khan, Rajesh N. Udavant

**Affiliations:** 1 Division of Plant Pathology, Faculty of Agriculture, Sher-e-Kashmir University of Agricultural Sciences and Technology of Jammu, Chatha, India; 2 Directorate of Research, Sher-e-Kashmir University of Agricultural Sciences and Technology of Jammu, Chatha, India; 3 ICAR-Central Institute of Temperate Horticulture, Srinagar, India; 4 Tamil Nadu Agricultural University, Coimbatore, India; 5 Department of Food Science and Nutrition, Faculty of Food and Agricultural Sciences, King Saud University, Riyadh, Saudi Arabia; 6 Department of Entomology, University of Georgia, Tifton, GA, United States

**Keywords:** metabolomic profiling, bioactive compounds, ragi, nutraceutical, phenolics

## Abstract

**Introduction:**

Finger millet (*Eleusine coracana*) is gaining increasing recognition as a functional food and a promising source of nutraceuticals for mitigating metabolic disorders, owing to its abundance of bioactive compounds. Despite its nutritional and therapeutic potential, comprehensive metabolomic profiling of its primary and secondary metabolites remains limited. This study aimed to perform an in-depth metabolomic analysis of finger millet landraces cultivated in the temperate region of Padder Valley, District Kishtwar, Union Territory of Jammu and Kashmir, and to assess the therapeutic relevance of these metabolites in preventing metabolic diseases.

**Methods:**

Comprehensive phytochemical profiling was conducted using liquid chromatography coupled with high-resolution tandem mass spectrometry (LC-HRMS/MS) to identify and characterize primary and secondary metabolites in finger millet grains. Inductively coupled plasma mass spectrometry (ICP-MS) was employed to quantify macro- and microelemental contents.

**Results:**

Metabolomic analysis identified a total of 50 primary metabolites, including derivatives of amino acids, fatty acids, and carbohydrates such as dehydroascorbic acid, niacin, xanthine, orotic acid, nicotinuric acid, gluconic acid, propionic acid, decanoic acid, and palmitic acid. Additionally, 135 secondary metabolites were characterized, encompassing heterocyclic compounds, phenolics, flavonoids, alkaloids, and terpenes such as 4-hydroxycyclohexylcarboxylic acid, 2-furoic acid, methyl cinnamate, mesitol, 4-hydroxybenzoic acid, heptalactone, viburtinal, and geranic acid. Elemental analysis revealed the presence of 10 macro- and microelements, with magnesium (Mg), calcium (Ca), potassium (K), and phosphorus (P) being the most abundant.

**Discussion:**

The comprehensive metabolite profiling demonstrates that finger millet is a rich source of bioactive primary and secondary compounds with potential therapeutic benefits. The diversity of metabolites and essential minerals highlights its value as a functional food ingredient for the prevention and management of metabolic disorders. These findings provide a biochemical basis for the development of value-added nutraceutical products derived from finger millet landraces.

## Highlights

- 53 phenolic compounds have been identified for the first time in finger millet in Jammu and Kashmir.
- Coumarin, and eugenol is the major phenolic compound in finger millet.
- Phenolic compounds significantly contribute to the antioxidant capacity.

## Introduction

Food insecurity and malnutrition are serious health problems in developing countries especially India and underdeveloped countries that impact millions of people. Currently, over 1.4 billion people are living in the country which has been increasing progressively, it is critical to address these issues to ensure nutritional equity and self-sufficiency (Baduni et al., 2024). Being the world’s primary producer of millet, India has a lot of potential to combat malnutrition and food insecurity. Millets, belong to the Poaceae family of small-seeded edible grasses and are found in arid and marginal areas of both tropical and temperate climates (Yadav et al., 2024). India accounts for 41% of the world’s production of millet, according to global data. (Vishal et al., 2024). Millets significantly improve nutritional and food security and increase genetic diversity of the global food basket (Mallick et al., 2024). Millets such as pearl millet (*Pennisetum glaucum*), foxtail millet (*Setaria italica*), porso millet (*Panicum miliaceum*), finger millet (*Eleusine coracana*), barnyard millet (*Echinochlo aesculenta*), kodo millet (*Paspalum scrobiculatum*), sorghum (*Sorghum bicolor*), and little millet (*Panicum sumatrense*) are rich in vital nutrients, gluten-free and low glycaemic index, to make them ideal for people with diabetes, degenerative, and coeliac diseases (Singh et al., 2024). As staple grains for centuries, millets played a significant role in Indian diets, especially in hilly and rural areas due to their low water requirement, drought tolerance, resistance to harsh weather conditions, climate resilience and also reduced input requirement (Vikash et al., 2024). However, with the advent of modern agricultural practices and the widespread cultivation of rice and wheat millet usage has declined sharply, contributing less than 10 per cent to the human diet (Ghosh et al., 2024).

*Eleusine coracana*, sometimes referred to as ragi, nachni, kodra or finger millet, is widely grown in China, India, and other parts of Africa. The highlands of Eastern Africa, especially Ethiopia and Uganda, are believed to be its birthplace (Tamilselvan et al., 2023). About 5,000 years ago, *E. coracana* was domesticated and then spread to Asia and other parts of Africa. It has the efficacy for addressing food security and malnutrition because of its high content of nutrition. Due to significant amounts of minerals, protein, carbohydrates, and dietary fiber, finger millet has a well-established nutritional value that draws a lot of interest in the present era (Deme et al., 2019). Additionally, micro and macronutrients, is a virtuous source of phytoconstituents, especially phenolic compounds, which help in lowering chronic diseases including cardiovascular diseases, diabetes, and cancer. Proanthocyanidins, hydroxybenzoic (p-hydroxybenzoic and, protocatechuic) acids, flavonoids (apigenin, quercetin, epicatechin, and catechin), and hydroxycinnamic (p-coumaric acid, ferulic acid) are predominant polyphenols present in finger millet (Kalsi et al., 2023). Finger millet has been identified as a prominent source of minerals and protein, although little is known about its phytochemical makeup. Understanding finger millet’s phytochemicals and nutritional value may enhance its application as a source of functional food material and help comprehend the associated health benefits (Abioye et al., 2022). In humans, severe chronic diseases include heart disease, cancer, diabetes, and cognitive dysfunction have been linked to the oxidation of cellular molecules by reactive species (Sharma et al., 2023). Dietary antioxidants play a crucial role in protection against oxidative damage and maintaining a healthy metabolic balance. Recent research has increasingly concentrated on plant bioactive compounds due to their several health benefits (Pandey et al., 2023).

Metabolomics as a relatively new field, is capable of analyzing every low molecular weight molecule present in particular organism or tissue. Liquid Chromatography Mass Spectrometer (LC-MS) technology has been employed in several research to detect and assess expression levels of various metabolites. These studies span multiple dimensions, including plant development (Theodoridis et al., 2023), plant-microbe interactions (Spina et al., 2021), human diseases (Zhou and Zhong, 2022), and plant nutrition (Zhong et al., 2022).

The phytochemical composition of *Eleusine coracana* remains inadequately explored in existing literature, highlighting the need for a comprehensive analysis of its beneficial compounds. In the current investigation, phytochemical analysis was conducting utilizing LC-MS/MS, while elemental analysis was performed through ICP-MS. The goal of this study was to examine the profiling of phytochemical compounds present in the local landraces of finger millet cultivated in the temperate region of District Kishtwar (Padder Valley) within the Union Territory of Jammu and Kashmir. The development of innovative food products and nutraceutical applications could benefit from an enhanced comprehensive of phytochemical components. This strategy may also contribute to the sustainable advancement of the Indian biome by harnessing the untapped potential of this underutilized crop and promoting biodiversity conservation. These findings of the present study offer a thorough and understandable explanation of the phytochemical and nutritional components of finger millet as well as the metabolic pathways connected to the metabolic compounds that were identified through differential analysis.

## Materials and methods

### Plant material

**Figure 1:**
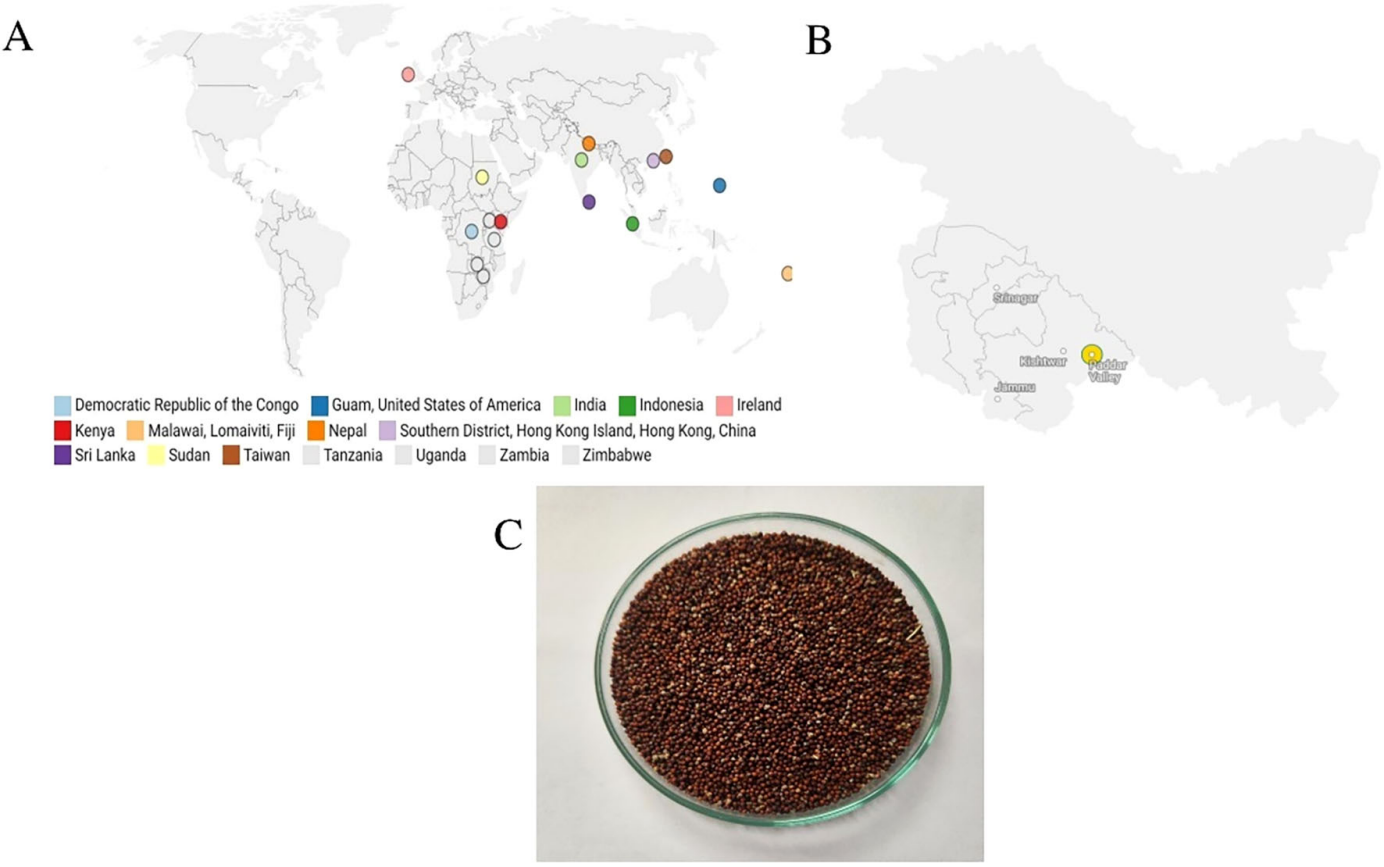
**(A)** Global distribution of finger millet **(B)** Local habitat for collection of finger millet in the UT of Jammu and Kashmir used in current study, **(C)** samples of finger millet seeds from Chasoti village of Padder Valley (District Kishtwar), Jammu and Kashmir, India.

Seeds of finger millet were collected from local habitat found in Chasoti village (33°34′ N latitude, 75°53′58″ E longitude, at an altitude of 1638 m), of Padder Valley (District Kishtwar), Jammu and Kashmir, India (**Figure 1**). The collected specimens were transported to Division of Plant Pathology, Faculty of Agriculture, SKUAST Jammu, within 48 hours of collection. Further, the samples were thoroughly rinsed with distilled water to eliminate soil particles and extraneous materials. The seeds were subsequently separated manually for further processing and analysis.

### Characterization of finger millet

Molecular characterization of the collected finger millet samples was performed to ensure precise validation. The seeds were germinated under controlled conditions in plant growth chamber on 30°C with 55% humidity and 12 h light, and the resulting seedling tissues were utilized for genomic DNA extraction to facilitate further molecular analyses (Ibrahim et al., 2023). Purified DNA is used as the template for PCR to amplify segments of *matK* and *rbcL* gene sequences. The primers *matK-F* (5′-CTTTGCATTTATTACGGCTC-3′), *matK-R* (5′-GATTGGTTACGGGAGAAAAAG-3′) and *rbcL-F* (5′-GAAGTAGTAGGATTGATTCT-3′), *rbcL-R* (5′-CATCATTATTGTATACTCTTTC-3′) were used to amplification (Molla et al., 2023). The PCR mix (20 µl), which included 10 µl of master mixture, 1 µl of 10 nm each primer, 1 µl of DNA, and 7 µl of milli Q, was supplemented with total DNA (50–500 ng). The following circumstances was performed when the reaction was carried out: 1 min of 94 °C, followed by 32 cycles for 10 sec at 98°C, 56°C and 55°Cfor 15 sec, 68°C for 30 sec, and final elongation of 3 min at 68°C. 1.5% agarose gel electrophoresis was used to isolate and visualise the amplified DNA products under UV light (**Supplementary Figure S1**). Biologia Research India Pvt Ltd., Karnal, Haryana sequenced the purified partial amplicons. Before being compared to those in the GenBank database using the Basic Local Alignment Search Tool (https://blast.ncbi.nlm.nih.gov/Blast.cgi), the sequences were constructed, altered, and aligned in MEGA11 to determine the sequence homology with closely related taxa. In this research, the plant species exhibiting the highest sequence identity (100%) were selected as closest taxonomic match for molecular identification (Almutairi, 2021).

### Preparation of extract

**Figure 2:**
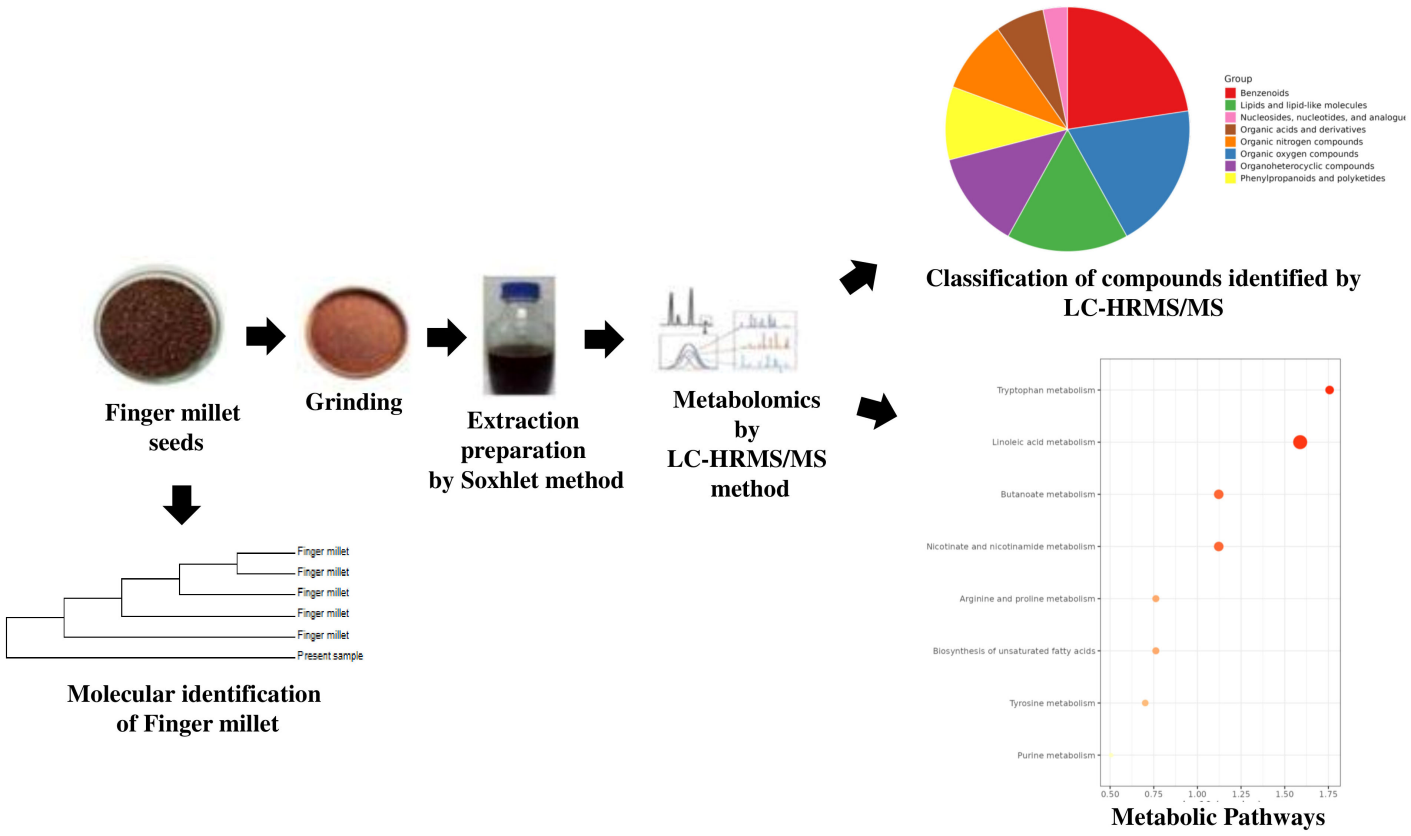
Workflow of LC–HRMS based untargeted metabolomic analysis of finger millet.

Finger millet dried seeds were coarsely grounded for extraction. 50 g of the powered seed sample from three independent seed batches (n = 3) was placed in a cellulose thimble and extracted using 400 mL of 95% methanol from Merck (Darmstadt, Germany) (65°C) in a Soxhlet apparatus. Exhaustive extraction was applied with solvent for 10–12 hours to ensure a complete extraction procedure (**Figure 2**). The extract was concentrated at 40°C under reduced pressure by using a rotary evaporator and was stored at 4°C until further use (Malathi et al., 2024).

### Elemental composition by ICP-MS

0.5-1.0 gm of homogenized powder of sample was weighed using weighing paper and transferred into polytetrafluoroethylene-Teflon (PTFE-TFM) vessels, performed in triplicate. Add 15 mL dilute nitric acid 70% (v/v), was purchased from Merck (Darmstadt, Germany) into the same vessel. The pre-digestion reactions were allowed to proceed for 20 mins. Then, the vessels were tightly closed and inserted into the 20SVT50 rotor. The rotor was subjected to Multiwave 5000 radiation system at 140°C for 30 mins. After completion of the digestion, the vessels were taken out of the microwave and were allowed to cool down to room temperature (25 °C) for 20 mins. The digests produced were quantitatively transferred into 25 mL volumetric flasks and the flasks were then filled up to mark with ultrapure water. The aqueous solutions were then passed through polyvinylidene difluoride (PVDF) microfilters, prior to metal analysis by ICP-OES. Each sample was digested in triplicates with a blank as the fourth sample (Mafukata et al., 2024).

### Liquid chromatography-high resolution mass spectrometry identification

LC-HRMS was used to identify secondary metabolites in the methanol extract of finger millet, using a previously described method with some modifications (Anggraeni et al., 2025). This study represents a preliminary profiling of finger millet. The analysis was performed in three independent replicates to ensure consistency. Biologia Research India Pvt Ltd., Karnal, Haryana performed the LC-HRMS/MS analysis. Extracted metabolite samples were analyzed for identifying and relatively quantifying using LC-HESI-MS/MS method on Vanquish Flex UHPLC coupled with Orbitrap Fusion™ Lumos™ Tribrid™ MS from Thermo Scientific. The polar metabolites were eluted by running the samples on Hypersil GOLD VANQUISH column (150mm x 2.1mm; 1.9 µ) column at column temperature of 40 °C. For UHPLC run, 0.1% formic acid in water and 0.1% formic acid in acetonitrile were used as mobile phase A and mobile phase B, respectively. The gradient run includes %B from 1 to 25 in 3.5 minutes, 25 to 35 in 4 minutes, 35 to 95 in 2.8 minute, a stable 95% for 3.7 minutes and re-equilibration at 1% for 6 minutes. The H-ESI was used for ionization at static spray voltage of 3400 (v) for positive ions. The full scans and MS2 scans were acquired at 120000 at scan range 50–1500 m/z. The ddMS2 spectra were acquired using Orbitrap detector at 60000 resolutions with HCD fragmentation using stepped collision energy at 20, 40, 75%. The raw spectral data were processed using Compound discoverer 3.3.2.3.1 Software and mzCloud server and ChemSpider database search.

### Kyoto encyclopedia of genes and genomes annotation and metabolic pathway analyses of differential identified compounds

Identified metabolites were annotated using the Kyoto Encyclopedia of Genes and Genomes (KEGG) compound database (http://www.kegg.jp/kegg/compound/) and annotated metabolites were mapped to the KEGG Pathway database (http://www.kegg.jp/kegg/pathway.html) using software MetaboAnalyst. Pathways with significantly regulated metabolites were then fed into metabolite set enrichment analysis, and their significance was determined by hypergeometric test p-values (**Supplementary Tables S1**, **S2**). The KEGG pathways with corrected *p*-values of less than 0.05 were considered significantly enriched by differentially expressed genes (Cao et al., 2022; Han et al., 2024).

### Statistical analysis

Every experiment was conducted three times, and the means of the results are provided. To compute the mean and standard deviation, SPSS-22 statistical software (SPSS, Inc., Chicago, IL, USA) was used.

## Results

### Molecular identification

**Figure 3:**
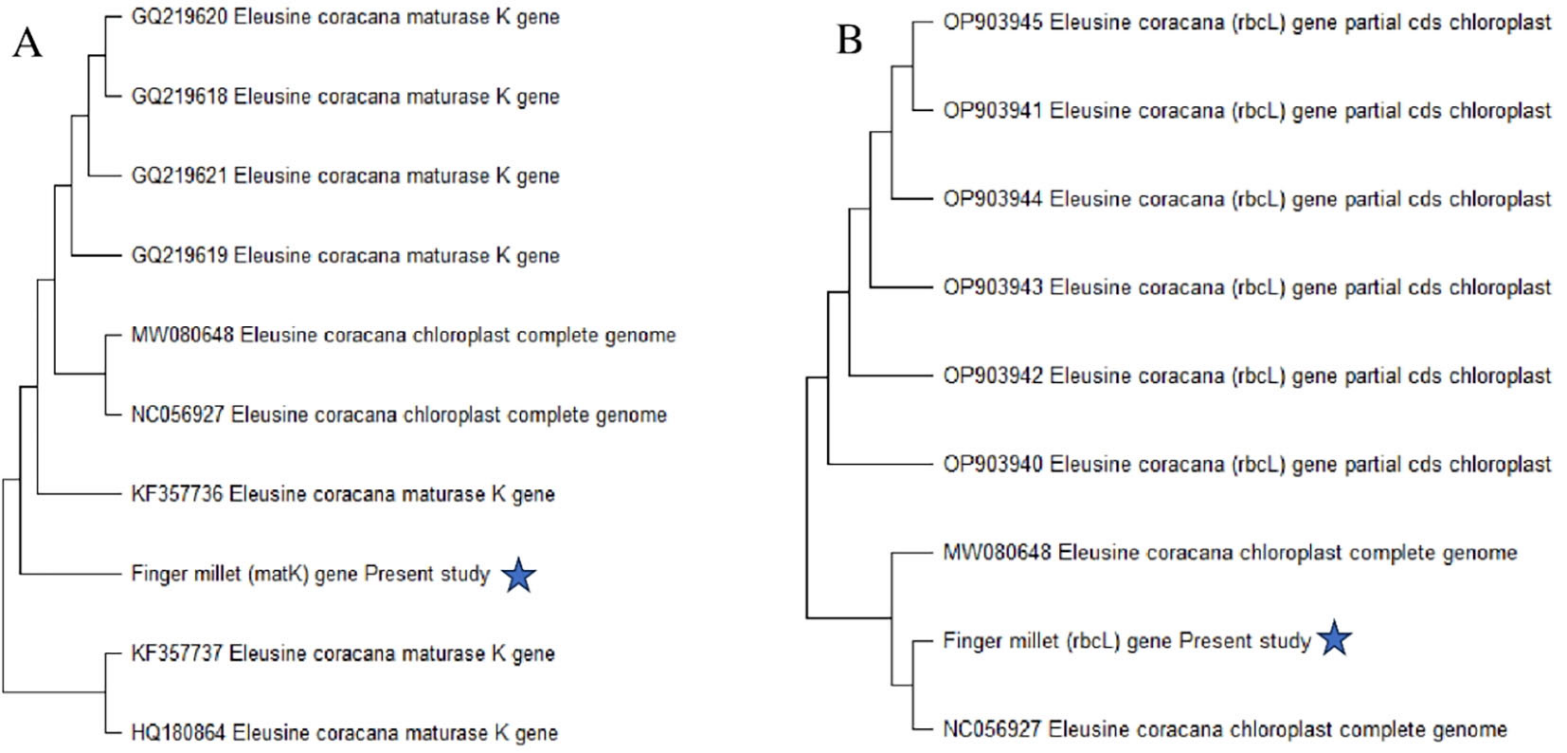
Maximum likelihood phylogenetic tree on basis of matK and rbcL sequence of *Eleusine coracana:***(A)** depicts phylogenetic tree on basis of matK and **(B)** depicts according to rbcL sequence of *Eleusine coracana*.

The *matK* and *rbcL* gene sequencing was utilized to identify finger millet sample, and a phylogenetic tree was made using MEGA 11 software. The Tamura 3-parameter model with lowest BIC and highest AIC values served as the basis for the maximum likelihood tree of the present millet sample, which was built using MEGA 11 and based on the study of *matK* and *rbcL* gene sequences. Every area with lacking information and gaps was eliminated. With gaps filled by pairwise deletion, the estimated transition/transversion bias (R) was 2.2. The bootstrap consensus tree was inferred from 1,000 to 3,000 iterations, and the maximum likelihood approach was used to reconstruct the evolutionary history. *Eleusine coracana*, the current millet sample, is well depicted in the dendrogram (**Figure 3**) that illustrates the evolutionary relationship. The sequences utilized in this investigation have been added to GenBank with accession numbers PQ753526 (*rbcL*) and PQ728252 (*matK*).

### ICP-MS analysis

Elemental analysis was carried out for the finger millet. A total of 10 macroelements and microelements were present at different concentrations (**Table 1**). The major elements found were calcium (Ca), potassium (K), phosphorus(P), and magnesium (Mg). Among all the analyzed elements, potassium was found to have the highest concentration. It is an essential mineral for the efficient functioning of tissues, the body’s cells, and organs. Increased potassium intake in diets can help maintain healthy blood pressure levels. The elements that are present in low concentrations are manganese (Mn), copper (Cu), iron (Fe), zinc (Zn), sodium (Na), and selenium (Se).

**Table 1:** ICP-MS analysis of finger millet.

S. No.	Minerals	Sample concentration (mg/100gram)
1	Ca	326.17 ± 0.8
2	Mg	138.24 ± 0.2
3	Zn	2.62 ± 1.4
4	Mn	2.13 ± 0.4
5	Fe	3.73 ± 0.4
6	Cu	0.67 ± 0.8
7	Se	0.04 ± 0.6
8	P	249.47 ± 0.8
9	K	416.93 ± 0.2
10	Na	1.27 ± 0.2

### Phytochemical profile of *Eleusine coracana* by LC-HRMS/MS

A developed and broad untargeted metabolomics method was conducted to the comprehensive phytochemical profile investigation of *Eleusine coracana* seed metabolites utilizing LC-HRMS/MS. Peaks were examined manually for signal/noise (s/n) > 10 in resulting data matrix, which included around 4000 signals with an MS2 spectrum overall. Duplicate signals were then eliminated as previously mentioned. Ultimately, the product ion spectra (MS2) yielded 521 very reproducible metabolite signatures. 185 were allegedly detected with the commercial standards based on fragmentation pattern, retention time (RT), and mass-to-charge-ratio (m/z) values of each metabolite. Aside from a few primary metabolites, the results included 13 fatty acid derivatives, 19 amino acids and their derivatives, 6 nucleotide derivatives, 7 carbohydrate derivatives, and 5 vitamin-related substances. An overview of all annotated compounds is given in **Tables 2**, **3**. Our study also putatively identified several secondary metabolites, including 53 phenolic compounds, 5 flavonoids, 13 terpenoids, 10 alkaloids, 13 heterocyclic compounds, 24 organic compounds, 6 ester compounds, 5 sugar alcohols, 4 quinoline compounds, and 2 acid derivatives. Significantly, in case of primary metabolites, four vitamin biosynthesis-related metabolites, including dehydroascorbic acid, niacin, vitamin C, D-pantothenic acid, 5′-O-β-D-glucosyl pyridoxine; various nucleotide derivatives including xanthine, orotic acid, nicotinuricacid, epiguanine, 7-carboxy-7-deazaguanine, allantoin; carbohydrate derivatives such as gluconic acid δ-lactone, methyl α-D-mannoside, xylitol, 2-deoxyhexopyranose; various fatty acid derivatives such as propionic acid, decanoic acid, palmitic acid, 6-oxohexanoic acid, triacetic acid, 6-oxohexanoic acid were detected in the seeds of *Eleusine coracana.*

**Table 2:** Secondary metabolite phytochemical profiling of the *Eleusine coracana* seed extract analyzed by LC-HRMS/MS.

Name	RT [min]	m/z	Molecular weight	Molecular formula	Delta mass [PPM]	Activity
Sugar alcohols						
Volemitol	5.18	211.082	212.089	C_7_ H_16_ O_7_	1.5	Antioxidant
D-(-)-Mannitol	5.07	181.072	182.079	C_6_ H_14_ O_6_	1.88	Antioxidant
D-(+)-Arabitol	4.77	151.061	152.068	C_5_ H_12_ O_5_	2.43	Antibiotic
1-Deoxy-L-mannitol	3.30	165.077	166.084	C_6_ H_14_ O_5_	2.64	Antioxidant
6-Methyltetrahydro-2H-pyran-2,5-diol	8.41	287.148	132.079	C_6_ H_12_ O_3_	4.33	–
Quinoline						
Xanthurenic acid	1.28	206.045	205.038	C_10_ H_7_ N O_4_	2.77	Neuroactive
Hydroxy-1,4-benzoquinone	1.80	123.009	124.016	C_6_ H_4_ O_3_	4.58	Cytotoxic
Hydroxynaphthoic acid	7.74	377.104	188.048	C_11_ H_8_ O_3_	3.84	Allelopathic
Benzoquinone	0.68	167.035	108.021	C_6_ H_4_ O_2_	3.83	Antimicrobial
Phenolic aldehyde						
β-Resorcylaldehyde	0.54	137.024	138.032	C_7_ H_6_ O_3_	2.98	Anti-inflammatory
Benzaldehyde	2.28	276.100	106.042	C_7_ H_6_ O	4.38	Antimicrobial
3-(4-Hydroxyphenyl)-2-oxiranecarbaldehyde	0.54	163.040	164.047	C_9_ H_8_ O_3_	1	–
Phenolic compounds						
Vanilpyruvic acid	3.50	209.045	210.053	C_10_ H_10_ O_5_	1.86	–
Vanillin	0.53	151.040	152.047	C_8_ H_8_ O_3_	2.62	Antioxidant
Urolithin B	8.91	447.085	212.047	C_13_ H_8_ O_3_	1.95	Anti-inflammatory
Umbelliferone	0.74	161.024	162.032	C_9_ H_6_ O_3_	2.33	Antioxidant
Scopoletin	0.78	191.035	192.042	C_10_ H_8_ O_4_	2.18	Antioxidant
Schisandrin C	12.17	385.166	384.158	C_22_ H_24_ O_6_	4.03	Antioxidant
Salicylic acid	0.74	137.024	138.032	C_7_ H_6_ O_3_	2.99	Antimicrobial
Protocatechuic acid	0.72	153.019	154.027	C_7_ H_6_ O_4_	2.93	Antioxidant
Phloroglucinol	4.08	125.024	126.032	C_6_ H_6_ O_3_	4.42	Phloroglucinol
Phenylpropiolic acid	1.80	145.030	146.037	C_9_ H_6_ O_2_	4.08	Anticancer
Phenylglyoxylic acid	0.49	149.024	150.032	C_8_ H_6_ O_3_	2.93	–
Phenylethyl alcohol	0.66	181.087	122.073	C_8_ H_10_ O	3.65	Antimicrobial
Phenol	0.73	93.034	94.042	C_6_ H_6_ O	3.32	Antioxidant
p-Cresol	0.38	107.050	108.057	C_7_ H_8_ O	3.2	Antimicrobial
Naphthalene-2,3-diol	0.94	159.045	160.05	C_10_ H_8_ O_2_	2.49	–
N-[2-Hydroxy-4(sulfooxy)phenyl]acetamide	6.15	145.028	247.015	C_8_ H_9_ N O_6_ S	0.85	–
Methyl cinnamate	1.79	161.061	162.068	C_10_ H_10_ O_2_	4.15	Antimicrobial
Mesitol	0.66	135.081	136.089	C_9_ H_12_ O	3.17	Antioxidant
Meconic acid	0.35	198.988	199.996	C_7_ H_4_ O_7_	2.22	–
Lithospermoside	6.99	330.119	329.111	C_14_ H_19_ N O_8_	1.57	Antioxidant
Isovanillic acid	0.41	167.035	168.042	C_8_ H_8_ O_4_	3.18	–
Hydroquinone	0.73	109.029	110.037	C_6_ H_6_ O_2_	3.86	–
Homovanillic acid	0.81	181.051	182.058	C_9_ H_10_ O_4_	2.24	–
Guaietolin	0.65	211.098	212.105	C_11_ H_16_ O_4_	2.79	Antimicrobial
Guaiacol	0.94	123.045	124.052	C_7_ H_8_ O_2_	3.55	Antimicrobial
Eugenol	1.75	163.077	164.084	C_10_ H_12_ O_2_	3.82	Antimicrobial
Dihydrophloroglucinol	0.92	187.061	128.047	C_6_ H_8_ O_3_	3.56	–
Coumarin	0.71	145.030	146.037	C_9_ H_6_ O_2_	3.65	Antimicrobial
Benzoic acid	10.11	121.029	122.037	C_7_ H_6_ O_2_	1.8	Antimicrobial
Benzeneacetamide-4-O-sulphate	7.65	273.054	231.020	C_8_ H_9_ N O_5_ S	1.46	–
Aceturic acid	1.18	98.025	117.043	C_4_ H_7_ N O_3_	3.45	–
5-carboxyvanillic acid	0.35	211.025	212.032	C_9_ H_8_ O_6_	2.22	Antioxidant
4-Hydroxyphthalic acid	0.41	181.014	182.022	C_8_ H_6_ O_5_	3.38	–
4-Hydroxycyclohexylcarboxylic acid	1.12	189.077	144.079	C_7_ H_12_ O_3_	4.39	–
4-Hydroxybenzoic acid	1.12	137.024	138.032	C_7_ H_6_ O_3_	4.13	Antimicrobial
4-Hydroxy-3-nitrophenylacetic acid	0.54	196.025	197.032	C_8_ H_7_ N O_5_	2.64	–
4-Ethylguaiacol	0.49	151.076	152.084	C_9_ H_12_ O_2_	2.93	Antimicrobial
4,5-Dihydroxyphthalic acid	0.36	197.009	198.016	C_8_ H_6_ O_6_	2.18	–
3-Hydroxybenzoic acid	3.37	137.024	138.031	C_7_ H_6_ O_3_	1.24	Antimicrobial
3-(4-Hydroxy-3-methoxyphenyl)-2-methylpropanoic acid	0.48	209.082	210.089	C_11_ H_14_O_4_	2.72	–
3,4-Dihydroxyphthalic acid	0.43	197.009	198.016	C_8_ H_6_O_6_	2.44	–
3,4-Dihydroxyphenylglycol	7.21	171.064	170.057	C_8_ H_10_O_4_	0.44	Antioxidant
2-Furoic acid	0.98	111.009	112.016	C_5_ H_4_ O_3_	3.6	Antimicrobial
2,4-Dihydroxybenzoic acid	0.40	153.019	154.027	C_7_ H_6_ O_4_	3.23	Antimicrobial
1,2-Benzoquinone	0.53	153.019	108.021	C_6_ H_4_ O_2_	3.57	Cytotoxic
(E)-p-coumaric acid	2.63	163.040	164.047	C_9_ H_8_ O_3_	2.25	Antioxidant
(E)-Isoferulic acid	3.46	193.051	194.058	C_10_ H_10_ O_4_	2.26	Anti-inflammatory
(E)-Ferulic acid	0.70	193.051	194.058	C_10_ H_10_ O_4_	2.34	Antimicrobial
(2Z)-3-(3-Hydroxyphenyl)-2-methylacrylaldehyde	1.12	161.061	162.068	C_10_ H_10_ O_2_	3.89	–
(2E)-3-(3,4-Dimethoxyphenyl)acrylic acid	0.77	207.066	208.074	C_11_ H_12_ O_4_	2.38	–
Alkaloids						
Roquefortine L	12.01	202.590	403.166	C_22_ H_21_ N_5_ O_3_	4.98	Antimicrobial
Putaminoxin	0.59	211.134	212.141	C_12_ H_20_ O_3_	2.75	Antimicrobial
Naphthalen-2-amine	4.81	142.066	143.073	C_10_ H_9_ N	2.22	–
D-(-)-Quinic acid	5.60	191.056	192.068	C_7_ H_12_ O_6_	2.08	Antioxidant
Chaksine	13.61	473.286	450.297	C_22_ H_38_ N_6_ O_4_	3.92	–
6-hydroxypseudooxynicotine	8.87	452.227	194.105	C_10_ H_14_ N_2_ O_2_	0.47	–
4-O-α-D-Glucopyranosylmoranoline	6.45	326.144	325.137	C_12_ H_23_ N O_9_	1.14	–
3-Methyloxindole	0.67	146.061	147.068	C_9_ H_9_ N O	2.8	–
3-hydroxy-3-methyloxindole	0.96	162.056	163.063	C_9_ H_9_ N O_2_	2.57	–
2-pyridone	0.72	140.035	95.037	C_5_ H_5_ N O	4.45	–
Terpenoids						
γ-Heptalactone	1.14	127.076	128.084	C_7_ H_12_ O_2_	3.98	–
Viburtinal	0.71	159.045	160.052	C_10_ H_8_ O_2_	3.26	Antimicrobial
Tulipalin A	0.53	195.066	98.037	C_5_ H_6_ O_2_	2.96	Cytotoxic
Trans-geranic acid	0.47	167.108	168.115	C_10_ H_16_ O_2_	3.02	Antimicrobial
Maraniol	0.95	203.071	204.079	C_12_ H_12_ O_3_	2.67	–
Glycyrin	12.00	383.149	382.142	C_22_ H_22_ O_6_	2.37	Anti-inflammatory
Geranyl formate	12.81	183.137	182.130	C_11_ H_18_ O_2_	0.23	–
Geranyl acetate	0.47	195.139	196.146	C_12_ H_20_ O_2_	2.1	Antimicrobial
Gallic acid	0.53	169.014	170.021	C_7_ H_6_ O_5_	2.32	Antioxidant
DL-Mevalonic acid	0.43	129.056	148.073	C_6_ H_12_ O_4_	2.93	–
Abietatriene	13.80	271.241	270.234	C_20_ H_30_	0.3	–
6-Methylhept-5-en-2-one	0.48	185.118	126.104	C_8_ H_14_ O	3.93	–
4-Methyl-2-propyltetrahydro-2H-pyran-4-yl acetate	0.50	199.134	200.141	C_11_ H_20_ O_3_	2.52	–
Heterocyclic compounds						
Thiophene	0.46	82.996	84.003	C_4_ H_4_ S	3.04	Anti-inflammatory
Thiazole	0.40	83.991	84.998	C_3_ H_3_ N S	1.95	–
Quinolone	4.17	144.046	145.053	C_9_ H_7_ N O	3.87	Antibacterial
Pyrrole	2.97	66.035	67.042	C_4_ H_5_ N	2.09	–
Lumazine	1.12	163.026	164.034	C_6_ H_4_ N_4_ O_2_	3.94	Antimicrobial
Isoxazolin-5-one	10.10	84.009	85.016	C_3_ H_3_ N O_2_	1.44	Antimicrobial
Isoplumbagin	0.71	187.040	188.047	C_11_ H_8_ O_3_	3.3	Anticancer
Indole	1.83	116.051	117.058	C_8_ H_7_ N	4.55	–
Furoic acid	1.85	202.038	169.004	C_3_ H_7_ N O_5_ S	1.89	Antimicrobial
Furan	1.12	135.045	68.026	C_4_ H_4_ O	3.73	Anticancer
4,5-Dihydroxy-3-oxo-1-cyclohexene-1-carboxylic acid	0.66	171.030	172.037	C_7_ H_8_ O_5_	2.63	–
2-(Methylthio)benzothiazole	2.58	404.039	181.002	C_8_ H_7_ N S_2_	2.53	–
2-(3-Hydroxy-3,4,5,6-tetrahydro-1H-cyclopenta-furan-4-yl)-3-methoxy-3-oxopropanoic acid	9.65	243.087	242.079	C_11_ H_14_ O_6_	3.62	–
Organic compounds						
1-Acetylcyclohexyl acetate	10.18	183.102	184.110	C_10_ H_16_ O_3_	1.37	–
2,5-Dimethyl-4-ethoxy-3(2H)-furanone	1.15	215.093	156.079	C_8_ H_12_ O_3_	4.05	–
Propenal	0.84	55.019	56.026	C_3_ H_4_ O	2.88	–
Phenylacetaldehyde	1.66	119.050	120.057	C_8_ H_8_ O	3.32	–
Methyl Phenyl Disulfide	1.06	157.014	156.007	C_7_ H_8_ S_2_	3.84	–
Isatin	10.22	146.061	147.032	C_8_ H_5_ N O_2_	1.34	Antibacterial
Indole-3-carbidol	2.96	146.025	147.068	C_9_ H_9_ N O	2.63	–
Heptenal	0.59	171.103	112.089	C_7_ H_12_ O	3.63	Antimicrobial
Glyoxylic acid	5.08	133.014	74.0	C_2_ H_2_ O_3_	4.17	–
Furfuryl acetone	3.51	137.061	138.068	C_8_ H_10_ O_2_	2.06	Antimicrobial
Furfuranol	2.10	97.029	98.037	C_5_ H_6_ O_2_	3.94	Antimicrobial
Formylpyruvate	5.50	115.004	116.011	C_4_ H_4_ O_4_	2.67	–
Ethylpropyldisulfide	1.63	137.045	136.038	C_5_ H_12_ S_2_	1.8	Antifungal
Cumene	0.66	119.087	120.094	C_9_ H_12_	3.37	–
Butyronitrile	1.83	70.065	69.057	C_4_ H_7_ N	0.2	–
Acetophenone	0.46	119.050	120.057	C_8_ H_8_ O	3.06	–
4-Oxo-2-propylpentanoic acid	0.52	157.087	158.094	C_8_ H_14_ O_3_	2.16	–
4-Methylthio-4-methyl-2-pentanone	1.41	147.084	146.077	C_7_ H_14_ O S	3.16	–
4-Hydroxy-5-methyl-3-furanone	4.86	113.024	114.032	C_5_ H_6_ O_3_	3.14	–
4,7-Dihydroxycoumarin	0.50	179.033	178.026	C_9_ H_6_ O_4_	1.81	Anti-inflammatory
4,5,7-Trihydroxycoumarin	2.97	193.014	194.021	C_9_ H_6_ O_5_	2.39	Antimicrobial
3,4-Dimethylthiophene	2.04	113.042	112.035	C_6_ H_8_ S	4.88	–
2-methylcitric acid	4.96	205.035	206.043	C_7_ H_10_ O_7_	2.27	–
Dhurrin	7.01	334.09	311.100	C_14_ H_17_ N O_7_	0.31	–
Flavonoids						
Catechol	0.41	109.043	110.058	C_6_ H_6_ O_2_	4.47	Antioxidant
Flaviolin	0.46	205.012	206.083	C_10_ H_6_ O_5_	2.17	Antioxidant
Pyrogallol	1.18	125.058	126.063	C_6_ H_6_ O_3_	3.61	Antioxidant
Kumarone	0.76	161.073	162.023	C_11_ H_14_ O	2.13	Anti-inflammatory
Purpurogallin	0.47	219.048	220.093	C_11_ H_8_ O_5_	2.89	Antioxidant
Esters						
Tetrahydrofurfuryl acetate	0.67	143.071	144.079	C_7_ H_12_ O_3_	2.72	–
Methyl sorbate	1.14	125.061	126.068	C_7_ H_10_ O_2_	3.51	Antimicrobial
Ethyl levulinate	3.69	189.077	144.079	C_7_ H_12_ O_3_	3.51	Antioxidant
Ethyl benzoylacetate	0.60	191.071	192.079	C_11_ H_12_ O_3_	2.77	–
Benzyl formate	3.99	135.045	136.053	C_8_ H_8_ O_2_	4.2	–
Acevaltrate	11.25	241.107	480.200	C_24_ H_32_ O_10_	1.42	–
Acid derivatives						
Suberic acid	1.00	174.089	173.082	C_8_ H_14_ O_4_	2.93	–
Crotonic acid	0.50	86.037	84.024	C_4_ H_6_ O_2_	2.62	–
Hormones						
Indoleacetylaspartate	6.467	290.057	288.036	C_14_ H_14_ N_2_ O_5_	1.59	–

PPM is a widely accepted unit used in high-resolution mass spectrometry to express the mass deviation. This value reflects the instrumental mass accuracy, which is critical for high-confidence metabolite identification. A low Delta Mass (typically within ±5 PPM) is considered indicative of a high-confidence match.

**Table 3:** Primary metabolite phytochemical profiling of the *Eleusine coracana* seed extract analyzed by LC-HRMS/MS.

Name	RT [min]	m/z	Molecular weight	Molecular formula	Delta mass [PPM]
Carbohydrate derivatives					
δ-Gluconic acid δ-lactone	1.892	177.041	178.048	C_6_ H_10_ O_6_	3.46
Xylitol	4.297	151.061	152.069	C_5_ H_12_ O_5_	3.87
Methyl α-D-mannoside	3.563	193.072	194.079	C_7_ H_14_ O_6_	2.85
L-(-)-Malic acid	4.212	133.014	134.022	C_4_ H_6_ O_5_	4.02
Hydroxyacetone phosphate	5.239	152.996	154.003	C_3_ H_7_ O_5_ P	2.13
6-Deoxy-3-O-methyl-β-D-galactopyranose	4.163	177.077	178.084	C_7_ H_14_ O_5_	3.66
2-Deoxyhexopyranose	4.993	163.061	164.068	C_6_ H_12_ O_5_	2.45
Nucleotide derivatives					
Xanthine	3.782	151.026	152.033	C_5_ H_4_ N_4_ O_2_	2.79
Orotic acid	0.49	155.010	156.017	C_5_ H_4_ N_2_ O_4_	2.23
Nicotinuric acid	1.133	179.046	180.054	C_8_ H_8_ N_2_ O_3_	3.12
Epiguanine	4.002	164.058	165.065	C_6_ H_7_ N_5_ O	3.4
7-Carboxy-7-deazaguanine	2.081	193.037	194.044	C_7_ H_6_ N_4_ O_3_	3.23
(S)-(+)-allantoin	3.831	157.037	158.044	C_4_ H_6_ N_4_ O_3_	2.15
Vitamins					
Vitamin C	5.13	175.025	176.032	C_6_ H_8_ O_6_	2.42
Niacin	2.903	122.058	123.024	C_6_ H_5_ N O_2_	3.08
Dehydroascorbic acid	1.762	173.067	174.017	C_6_ H_6_ O_6_	3.6
D-pantothenic acid	12.238	439.230	219.111	C_9_ H_17_ N O_5_	2.22
5′-O-β-D-Glucosylpyridoxine	6.998	332.134	331.127	C_14_ H_21_ N O_8_	2.22
Amino acid derivatives					
N-Acetyl-L-phenylalanine	0.713	206.082	207.090	C_11_ H_13_ N O_3_	3.28
N-Acetyl-L-leucine	1.351	172.098	173.105	C_8_ H_15_ N O_3_	2.55
N-Acetyl-L-glutamic acid	7.051	379.135	189.064	C_7_ H_11_ N O_5_	4.63
N-Acetyl-D-quinovosamine	4.919	204.088	205.095	C_8_ H_15_ N O_5_	1.33
N-(3-Carboxypropanoyl)-5-hydroxynorvaline	7.223	467.188	233.090	C_9_ H_15_ N O_6_	1.19
Maleamic acid	0.509	114.020	115.063	C_4_ H_5_ N O_3_	3.14
L-Pyroglutamic acid	3.458	188.056	129.042	C_5_ H_7_ N O_3_	2.91
L-Proline	1.17	114.056	115.063	C_5_ H_9_ N O_2_	4.26
L-Histidinol phosphate	8.502	222.064	221.057	C_6_ H_12_ N_3_ O_4_ P	2.74
L-(−)-threo-3-Hydroxyaspartic acid	1.981	150.039	149.032	C_4_ H_7_ N O_5_	0.64
L-(+)-Aspartic acid	1.134	132.030	133.038	C_4_ H_7_ N O_4_	3.67
Isovalerylglycine	1.783	218.104	159.090	C_7_ H_13_ N O_3_	4.95
DL-Glutamine	4.999	145.062	146.032	C_5_ H_10_ N_2_ O_3_	2.87
D-(+)-Tryptophan	4.818	203.082	204.090	C_11_ H_12_ N_2_ O_2_	1.83
Aminolevulinic acid	1.125	130.051	131.058	C_5_ H_9_ N O_3_	4.04
γ-Thiomethyl glutamate	3	194.248	193.163	C_6_ H_11_ N O_4_ S	3.71
6-Acetamido-2-oxohexanoic acid	4.035	168.324	187.012	C_8_ H_13_ N O_4_	3.33
4-Methyleneglutamic acid	4.972	158.027	159.086	C_6_ H_9_ N O_4_	2.12
3-Sulfamoylalanine	8.028	186.034	168.063	C_3_ H_8_ N_2_ O_4_ S	2.37
Fatty acid derivatives					
Triacetic acid	4.947	143.035	144.042	C_6_ H_8_ O_4_	2.49
Sorbic acid	3.913	93.034	112.052	C_6_ H_8_ O_2_	3.39
Propionic acid	0.792	73.029	74.036	C_3_ H_6_ O_2_	2.41
Palmitic Acid	15.237	535.471	256.240	C_16_ H_32_ O_2_	0.11
Decanoic acid	10.209	171.139	172.146	C_10_ H_20_ O_2_	0.89
8-Hydroxy-5,6-octadienoic acid	0.624	215.093	156.079	C_8_ H_12_ O_3_	3.59
6-Oxohexanoic acid	0.883	189.077	130.063	C_6_ H_10_ O_3_	3.07
3E-octenoic acid	0.46	141.092	142.099	C_8_ H_14_ O_2_	2.46
3-Hydroxysebacic acid	0.811	217.108	218.115	C_10_ H_18_ O_5_	2.27
2-Hydroxyundecanoate	13.933	425.288	201.149	C_11_ H_21_ O_3_	3.5
1-Nonanoic acid	0.662	157.123	158.131	C_9_ H_18_ O_2_	2.35
1-Hexanal	0.531	99.081	100.089	C_6_ H_12_ O	3.5
9-Oxononanoic acid	0.719	171.103	172.110	C_9_ H_16_ O_3_	3.18

PPM is a widely accepted unit used in high-resolution mass spectrometry to express the mass deviation. This value reflects the instrumental mass accuracy, which is critical for high-confidence metabolite identification. A low Delta Mass (typically within ±5 PPM) is considered indicative of a high-confidence match.

The phenolic compounds in the seed were coumarin, guaiacol, eugenol, phloroglucinol, 4-hydroxycyclohexylcarboxylic acid, 2-furoic acid, 1,2-benzoquinone, 4-hydroxybenzoic acid, phenylpropiolic acid, methyl cinnamate, schisandrin C, mesitol, dihydrophloroglucinol. Similarly, terpenoids compounds detected in seeds of *Eleusine coracana* were γ-heptalactone, viburtinal, geranic acid, 6-methylhept-5-en-2-one, maraniol, gallic acid, glycyrinetc; alkaloids compounds including roquefortine L, 2-pyridone, chaksine, putaminoxin, 3-methyloxindole; phytohormone include indoleacetylaspartate; acid derivatives such as suberic acid and crotonic acid.

### Classification of metabolites detected using LC-MS/MS

**Figure 4:**
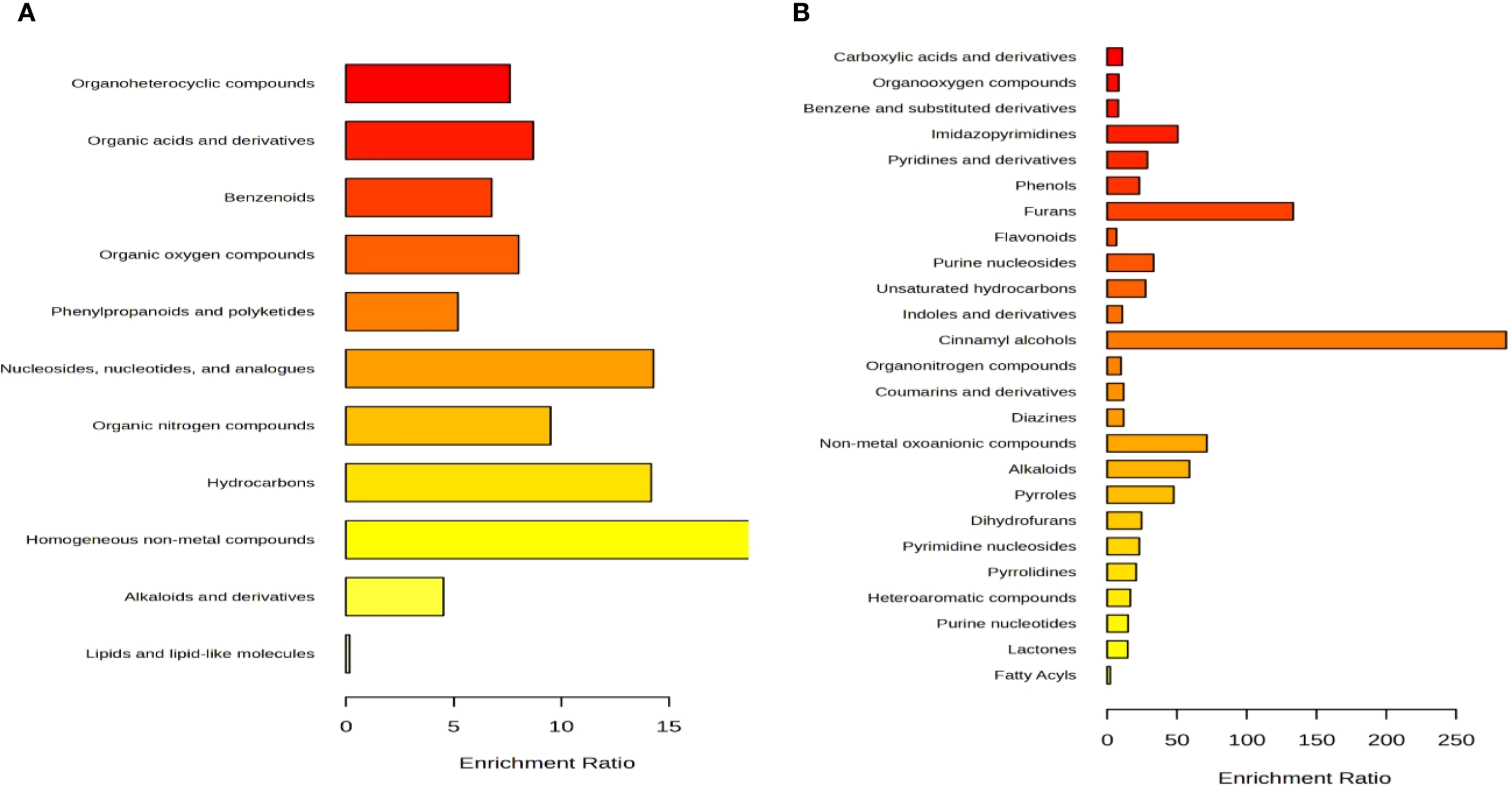
Detection and analysis of compounds according to LC–HRMS/MS. **(A)** indicate bar graph of the superclass of 185 differential identified metabolites; **(B)** indicate bar graph of the main class of 185 differential identified metabolites detected in the *Eleusine coracana* seed extract analyzed by LC-HRMS/MS.

Untargeted metabolomics of *Eleusine coracana* collected from the temperate region of District Kishtwar (Padder Valley), revealed putative compounds belonging to 9 different superclasses of compounds such as hydrocarbon derivatives, nitrogen, lipid, and oxygen compounds, heterocyclic compounds, phenylpropanoids, benzenoids, organosulfur compounds, organic acids and its derivatives (**Figure 4**). Further, detected metabolites were classified into 25 different main classes such as coumarin, cinnamic acid, indole and keto acid derivatives, fatty acyls, pyridine derivatives, furans, lactones, heteroaromatic compounds, carboxylic acid derivatives among others.

### KEGG annotation analysis of identified metabolites

**Figure 5:**
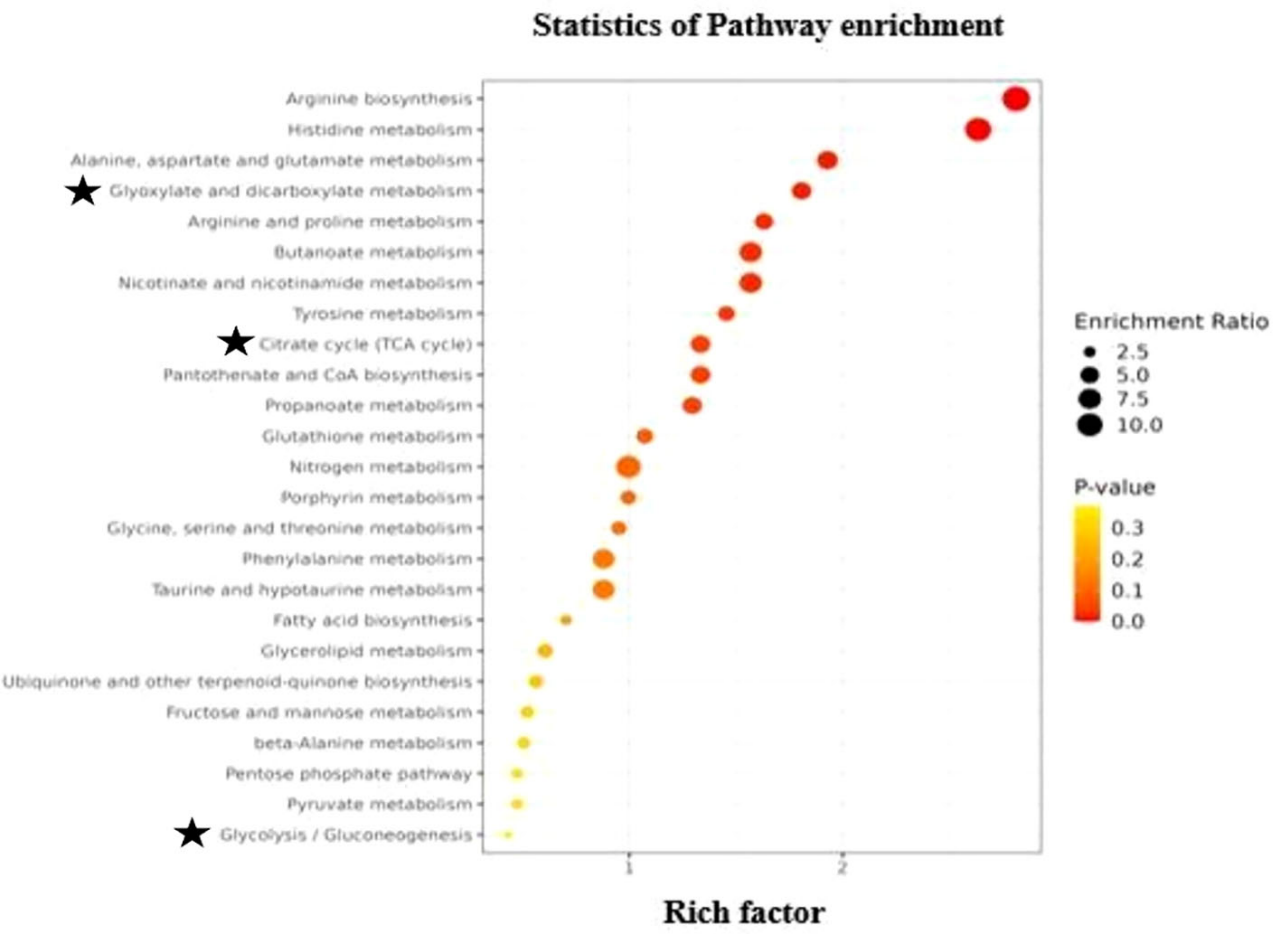
Scatter plot of top 25 KEGG pathways for differential identified metabolites in the seeds of *Eleusine coracana*. Abscissa, or rich factor, indicates the percentage of metabolites in a certain route that have been differentially identified. Circle color denoted ranges of corrected P value, while circle area showed number of metabolites that were differentially identified. The ratio of the number of metabolites in the associated pathway that were differentially identified to total number of metabolites that the pathway detected and annotated is known as the rich factor. The degree of enrichment increases with the value.

KEGG analyses were carried out to classify the functions of differentially identified metabolites and clarify biological pathways and functions in finger millet seeds. KEGG analysis results demonstrated that the metabolic pathways were mapped to identified metabolites in finger millet seed. Based on the findings presented in **Figure 5**, 185 differential metabolites were identified as participating in the biosynthesis of amino acids, carbohydrate metabolism, citric acid pathway, metabolism of nitrogen, glutathione and porphyrin, biosynthesis of fatty acid, terpenoid biosynthesis, metabolism of propanoate. Notably, pathways involved in carbohydrate metabolism highlighting the grain’s role as a rich source of complex carbohydrates. Additionally, enrichment in amino acid biosynthesis pathways underscores its protein content, while phenylpropanoid biosynthesis points to the presence of antioxidant compounds contributing to finger millet’s health-promoting properties. Phenylpropanoids help plants withstand pests and diseases, and research on Arabidopsis shows that the phenylpropanoid metabolic pathway is activated by salt and drought stress. Amino acid production is essential to an organism’s growth and development. Plants under LT stress typically had higher levels of free amino acids than control plants, suggesting that amino acid production responds favorably to stress (Pandohee et al., 2023). Research has demonstrated that in transgenic alfalfa lines containing myoinositol phosphate synthase (MIPS), abiotic stressors including cold, drought, and salt stressors cause IP1 to be generated, while H_2_O_2_ and NO stressors cause high expression of MIPS. In plant tissues, glutathione metabolism is essential for preserving antioxidant qualities and controlling redox-sensitive signal transduction (Yang et al., 2024). Plant tolerance to range of biotic and abiotic environmental stressors is directly correlated with glutathione levels (Ye et al., 2024).

## Discussion

The assessment of food and crop products as well as functional genomic research have made extensive use of metabolite profiling and chemometrics, which further guides breeding methods for enhancing and optimizing balance of food components (Li et al., 2021). Several metabolites can be identified and quantified in a single extract by applying the broad target metabolomics technique with LC-HRMS/MS (Dan et al., 2021). Metabolomics has been widely used to examine various compounds found in food products. Numerous significant metabolites with a range of physiological and bioactive roles have been identified by metabolomics investigation of several cereal crops, including millets (Wu et al., 2022). During the barnyard millet’s developmental stages, a total of 35 metabolites were found (Meniya et al., 2023). Sugar and sugar alcohols were most abundant of these compounds, followed by amino acids, organic acids, and sterols, in that order. Putative metabolites from 15 different groups of chemicals, including flavonoids, amino acids, organic acid and its derivatives, were identified by untargeted metabolomics of two distinct foxnut kinds that were gathered from various geographical locations (Bao et al., 2022). Numerous physiological effects on the human body, including antioxidative, antimicrobial, neuro-protective, anti-diabetic, antipyretic, anti-inflammatory, anti-depressive, anti-septic, antibiotic, anti-angiogenic, immunomodulatory activities, and anti-carcinogenic, have been demonstrated by number of these compounds that were identified in the current study (Saxena et al., 2024). Plant products include coumarin, which has been shown to have anti-inflammatory, antibacterial, antioxidant, and neuroprotective properties (Vishal et al., 2024). Pervious study observed that catechin and epicatechin were found to be the main polyphenols in all the finger millet samples (Xiang et al., 2019). Quinic acid is an alkaloid molecule with anti-inflammatory, antibacterial, and antioxidant properties that is found in plant metabolites (Deme et al., 2019). The current study also identified abietatriene, a diterpene with range of biological characteristics, such as anti-inflammatory, antibacterial, antiparasitic, antifungal, antioxidant, and antiviral actions (Kuźma and Gomulski, 2022). Results from several studies using animal models and cell cultures indicate that abietatriene has significant advantages by halting cellular DNA damage over time (Sena et al., 2024). Because of its flavour, glycyrin, also referred to as sugar alcohol, is frequently employed as a sweetener in the food sector. Furthermore, studies have shown that glycerin is useful as a skin protectant and to treat glaucoma (Pan et al., 2024).The antioxidant and anti-inflammatory potential of several phenolic compounds found in nutritional bar’s metabolome, including ferulic acid, guaiacol, eugenol, and guaietolin, have been thoroughly investigated (Pan et al., 2020).

Phenolic chemicals can withstand gastrointestinal digestion and stay intact as they pass through the colon because they are found in bound form in cereals, such as millet. These substances may have protective effects on the colon after being released by microbial fermentation (Sun et al., 2022). This phenomenon may help explain how whole grains can prevent colon cancer. Ferulic acid was found to significantly decrease production and expression of inflammatory markers include tumour necrosis factor-α (TNF-α), nitric oxide (NO), and interleukin-1β (IL-1β) while increasing levels of anti-inflammatory factor β-endorphin (β-EP) in a human HMC-3 microglial cell model (Vishal et al., 2024). Previous study showed that phenolic compounds such as syringic acid, hydroxyl caffeic acid, caffeic acid, coumaric acid ethyl ester, ellagic acid glucoside, 3-hydroxycoumarin, and feruloyl tartaric acid were identified in seed coat of finger millet (Soujanya et al., 2024). Antibacterial, anti-inflammatory, and antioxidant capabilities are mainly possessed by the hydroxybenzoic acid. Most fruits have a low hydroxybenzoic acid concentration, however, berries including blackberries, blackcurrants, strawberries, raspberries, and pomegranates can have very high levels of this compound (Carrillo et al., 2023; Kumar et al., 2024a). Coumarin, a naturally occurring phenolic compound identified in finger millet, exhibits multiple therapeutic activities. It acts as an anticoagulant by inhibiting vitamin K epoxide reductase, thereby reducing blood clot formation. Additionally, coumarin and its derivatives have been shown to possess anti-inflammatory and antioxidant properties by modulating inflammatory cytokines and scavenging reactive oxygen species (ROS), which are critical in the prevention of chronic diseases such as cardiovascular disorders and cancer (Sharma and Mallubhotla, 2022; Hussain et al., 2019; Kumar et al., 2024b). Eugenol, another key metabolite, is well known for its potent antimicrobial, analgesic, and anti-inflammatory effects. It exerts these effects primarily through the inhibition of COX-2 and NF-κB signaling pathways, which are involved in inflammation and pain signaling. Eugenol also disrupts microbial membranes, making it effective against a broad range of pathogens. Its antioxidant capacity further contributes to its role in protecting cells from oxidative damage, supporting its application in managing oxidative stress-related conditions (Ulanowska et al., 2021; Sharma et al., 2024).

Furthermore, flavonoids were identified as the primary phenolic compound class present in *Eleusine coracana* seeds by the LC-MS approach for phenolic compound quantification (Buelvas et al., 2021), the most abundant phenolic components in the crude extract of *Eugenia pollicina* leaves were flavonoids. Seeds of *Eugenia uniflora* primarily contain phenolics from the flavonoid class, including quercitrin and kaempferol pentoside (Fidelis et al., 2022). Both internal and external factors, including physical, chemical, and biological ones, affect the quantity of phenolic compounds. Moreover, the amount of total phenolic chemicals in plant tissues is similarly influenced by photosynthesis and carbon production (Araujo et al., 2021).

By analyzing the millet’s primary and secondary metabolites, we have been able to examine their detailed profiles and try to better understand their roles (Girardelo et al., 2020). The current study employed UHPLC-ESI-QTOF-MS, a potent, dependable, and combinative analytical approach, to quickly characterize a number of phytochemicals in extracts from two Lamiaceae plants (Cocan et al., 2018). It is evident from comparing our results with those of relevant literature that the phenolic compounds (gallic acid, ferulic acid) of porso and foxtail millets was comparable to that of the study suggested by (Pujari and Hoskeri, 2022). Thus, *Eleusine coracana* proved to be a prominent source of phenolic, flavonoid, and terpene chemicals, and its exploitation can find intriguing uses in pharmaceutical, cosmetic, functional food, food supplement, and novel product creation (Dey et al., 2022).

## Conclusion

This study provides a comprehensive nutritional and bioactive profiling of underutilized finger millet (*Eleusine coracana*) landraces from Padder Valley, District Kishtwar, Jammu and Kashmir, India. LC-HRMS/MS analysis identified key metabolites, including catechol, flaviolin, pyrogallol, ferulic acid, protocatechuic acid, coumarin, eugenol, cumene, and guaietolin, alongside significant fatty acids, alkaloids, terpenes, and hydrocarbons. These compounds exhibit diverse bioactivities, such as antioxidant, anti-diabetic, antibacterial, and anti-obesity effects, underscoring the potential of finger millet as a functional food ingredient and source of nutraceuticals. The findings highlight the crop’s capacity to contribute to dietary diversification, address chronic illnesses, and combat malnutrition. While the results demonstrate substantial therapeutic potential, they are based on metabolomic and in silico analyses. Therefore, future studies should focus on *in vivo* validation, mechanistic investigations, and targeted quantification to confirm the physiological relevance of these bioactive compounds and explore their applications in nutrition, health, and functional food development.

## Data Availability

The datasets presented in this study can be found in online repositories. The names of the repository/repositories and accession number(s) can be found in the article/**Supplementary Material**.
