# Supplementary material for: Metabolomic profiling of finger millet: unlocking the secrets of a nutritious staple food
Source: Front Plant Sci. 2025 Nov 6;16:1570787. doi: 10.3389/fpls.2025.1570787 (PMC12632812; doi:10.3389/fpls.2025.1570787)
Supplement: Supplementary file 1 [file SupplementaryFile1.pptx]

## Slide 1
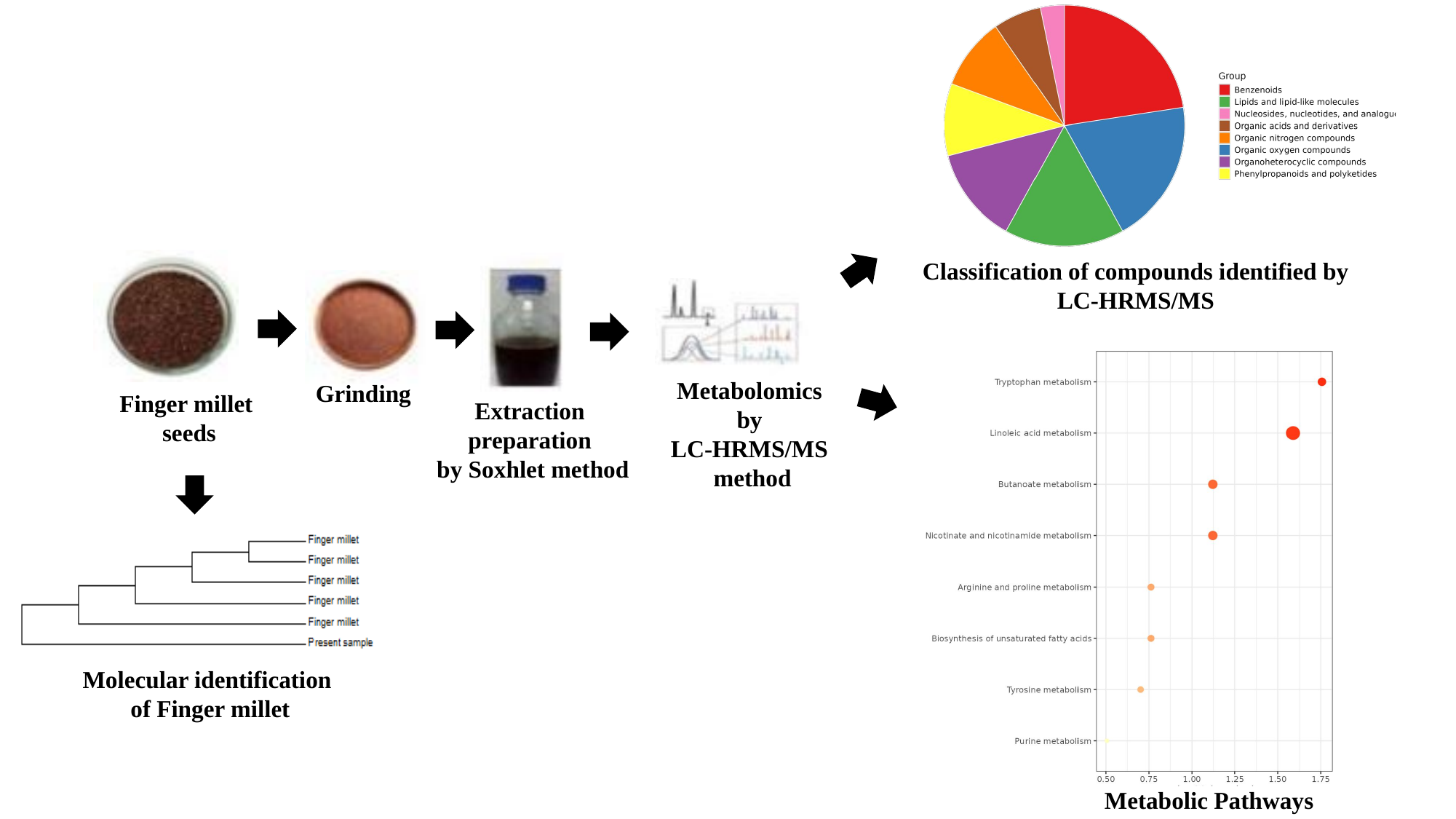

Classification of compounds identified by
LC-HRMS/MS
Metabolomics
by
LC-HRMS/MS
method
Grinding
Finger millet
seeds
Extraction
preparation
by Soxhlet method
Molecular identification
of Finger millet
Metabolic Pathways
